# Indispensable role of Mdm2/p53 interaction during the embryonic and postnatal inner ear development

**DOI:** 10.1038/srep42216

**Published:** 2017-02-09

**Authors:** M. Laos, M. Sulg, A. Herranen, T. Anttonen, U. Pirvola

**Affiliations:** 1 Division of Physiology and Neuroscience, Department of Biosciences, University of Helsinki, 00014 Helsinki, Finland

## Abstract

p53 is a key component of a signaling network that protects cells against various stresses. As excess p53 is detrimental to cells, its levels are tightly controlled by several mechanisms. The E3 ubiquitin ligase Mdm2 is a major negative regulator of p53. The significance of balanced p53 levels in normal tissues, at different stages of lifetime, is poorly understood. We have studied *in vivo* how the disruption of Mdm2/p53 interaction affects the early-embryonic otic progenitor cells and their descendants, the auditory supporting cells and hair cells. We found that p53 accumulation, as a consequence of *Mdm2* abrogation, is lethal to both proliferative progenitors and non-proliferating, differentiating cells. The sensitivity of postmitotic supporting cells to excess p53 decreases along maturation, suggesting that maturation-related mechanisms limit p53′s transcriptional activity towards pro-apoptotic factors. We have also investigated *in vitro* whether p53 restricts supporting cell’s regenerative capacity. Unlike in several other regenerative cellular models, *p53* inactivation did not alter supporting cell’s proliferative quiescence nor transdifferentiation capacity. Altogether, the postmitotic status of developing hair cells and supporting cells does not confer protection against the detrimental effects of p53 upregulation. These findings might be linked to auditory disturbances observed in developmental syndromes with inappropriate p53 upregulation.

p53 is well known for its role in guarding genomic integrity upon DNA damage and oncogene activation. Its tumor suppressor function is exerted through promotion of cell cycle arrest and apoptosis^1^. *p53* null mice are born alive and several of their tissues show an unaltered phenotype. Whether other p53 family members play a compensatory role remains an open question^2^,^3^. p53 is a short-lived protein. The key regulator maintaining its low levels in normal tissues is Mdm2. Mdm2 is an E3 ubiquitin ligase that promotes p53 degradation by the proteasome, but it also suppresses p53′s transcriptional activity^4^. Genetic disruption of Mdm2/p53 interaction leads to widespread induction of apoptosis and spontaneous lethality of early embryos^5^,^6^. Studies employing mouse models with conditional *Mdm2* ablation have shown that proper Mdm2/p53 interaction is needed for morphogenesis during mid- and late-embryogenesis as well^7^,^8^. Proper Mdm2/p53 interaction is also required in adult tissues, particularly in those containing high numbers of proliferating cells, shown both by inducible *Mdm2* ablation and by inducible, direct *p53* upregulation^9^,^10^,^11^.

Except for global examination of various tissues, only a few studies exist where the response of a given cell type to increased p53 stability has been comparatively studied in a continuum from development to adulthood, with the aim to dissect the importance of balanced p53 levels for cellular survival, differentiation and homeostasis. We have here addressed these questions in the proliferating progenitor cells of the embryonic inner ear and in one lineage of their descendants, the postmitotic epithelial supporting cells (SCs) of the auditory organ, at different stages of life. We have employed a conditional *Mdm2* mutant mouse model^12^ to disrupt Mdm2/p53 interaction *in vivo*. We have also pharmacologically disrupted Mdm2/p53 interaction *in vitro*.

In regeneration research, auditory SCs serve as a potential platform for the replacement of lost auditory sensory cells, the hair cells, by new ones. Many attempts have been made to trigger mammalian SCs to re-enter the cell cycle, to stimulate their transdifferentiation into hair cells, and to increase their stemness^13^. These studies have shown that while some degree of regenerative plasticity is retained in neonatal SCs, plasticity becomes very limited after this young age. Understanding the p53 response of auditory SCs with potential regenerative capacity is relevant, as p53 is known to antagonize regenerative interventions in various cellular contexts, best-known in restricting reprogramming of somatic cells into induced pluripotent stem cells^14^,^15^. Therefore, in addition to gain-of-function approaches, we have here studied whether *p53* inactivation confers regenerative plasticity to auditory SCs.

## Results

### Disruption of Mdm2/p53 interaction is detrimental to embryonic otic progenitor cells

By generating *Mdm2*^*FM*/*FM*^*;Pax2-Cre* mice, we first studied the response of otic placodal cells to the disruption of the p53 binding domain of Mdm2^12^. The ectodermal otic placode contains progenitor cells that give rise to the epithelial and neuronal compartments of the inner ear. Pax2 is one of the earliest markers of the otic lineage and it is broadly expressed in the otic placode^16^. In the *Pax2-Cre* mouse line, *Cre*-mediated recombination occurs in the early otic placode. Thereafter *Cre* mRNA expression is quickly downregulated, already at the late placodal stage^16^.

**Figure 1:**
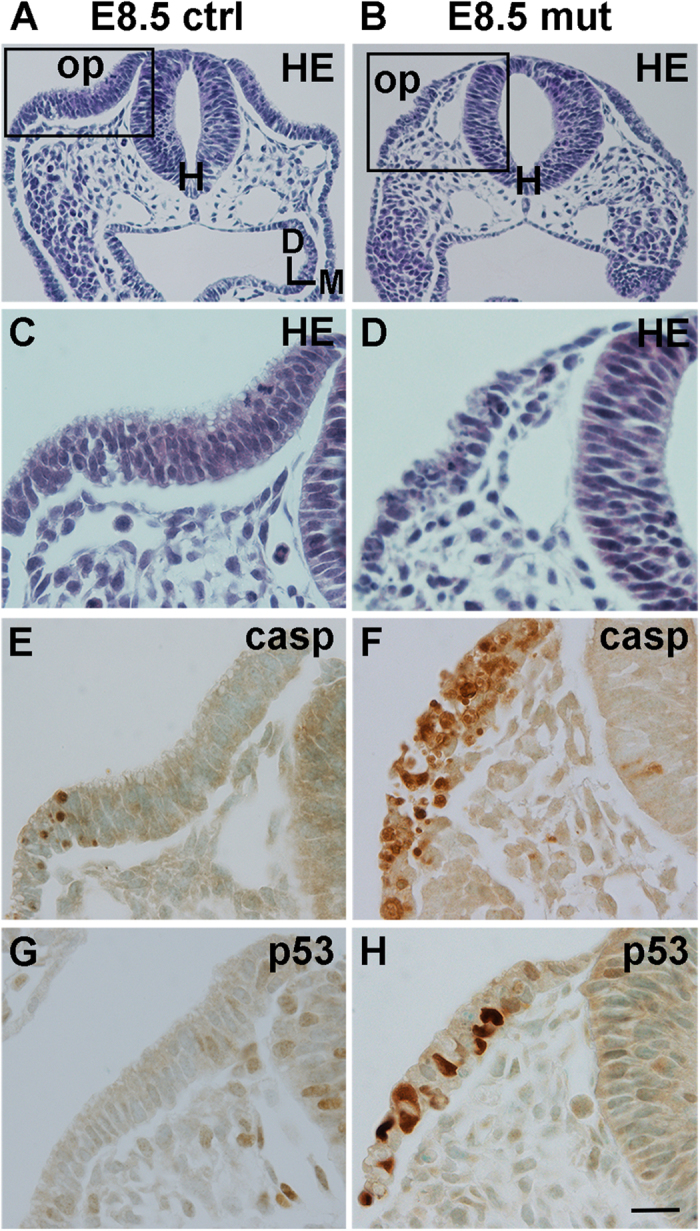
Disruption of Mdm2/p53 interaction triggers massive apoptosis in the otic placode, revealed in *Mdm2*^*FM*/*FM*^;*Pax2-Cre* mice. (**A–D**) At E8.5, hematoxylin-stained sections show that the otic placodes of mutant embryos are markedly reduced in size compared to controls. (**E,F**) While normal developmental apoptosis is seen at the edge of the control placode, cleaved caspase-3-positive progenitors accumulate in the mutant placode. (**G,H**) p53 is upregulated in the mutant, but not control placode. Abbreviations: D, dorsal; HE, hematoxylin; H, hindbrain; M, medial; op, otic placode. Scale bar, shown in **H:** (**A–H**), 20 μm.

**Figure 2:**
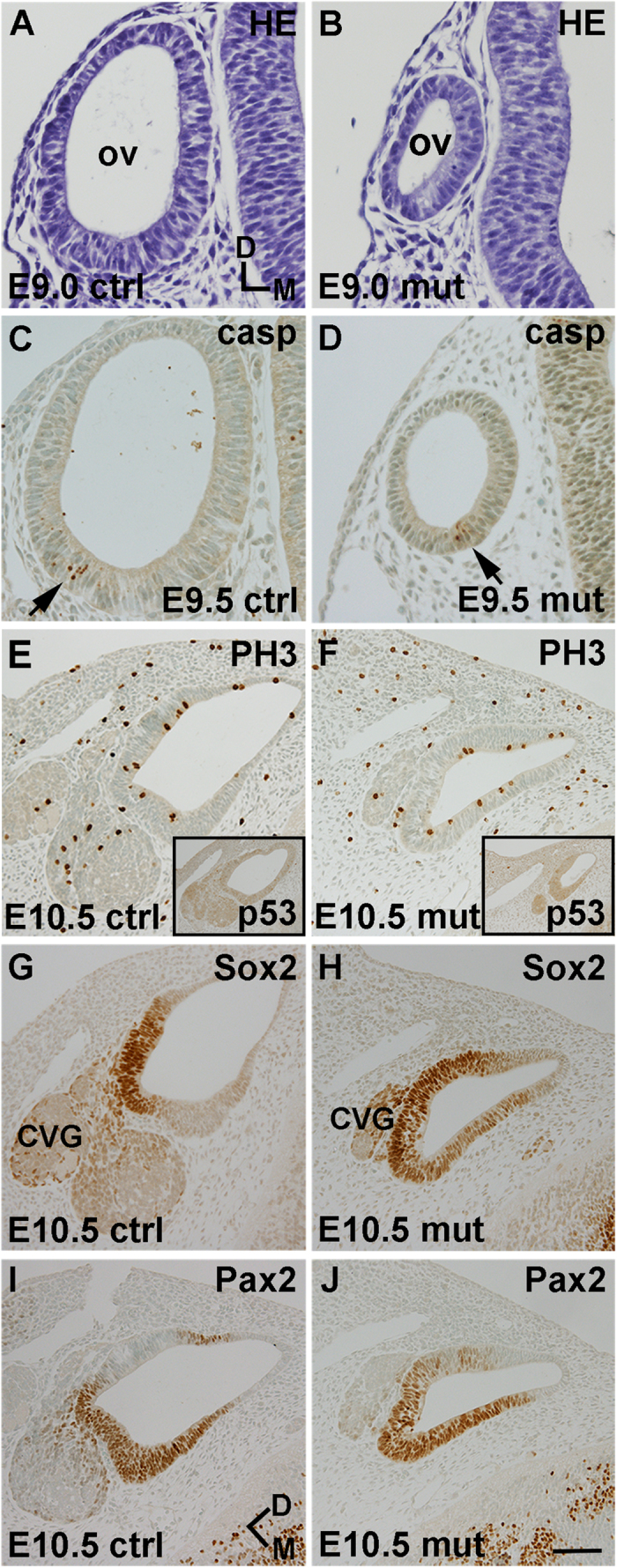
Morphogenesis is impaired in the early-developing inner ear of *Mdm2*^*FM*/*FM*^*;Pax2-Cre* mice. (**A,B**) At E9.0, otic vesicles of mutant embryos are smaller than those of controls. (**C,D**) At E9.5, the amount of cleaved caspase-3-positive cells (arrow) is comparable in mutant and control vesicles. (**E,F**) At E10.5, phosphorylated histone-3 staining shows mitotic activity at the lumenal surface of both control and mutant otocysts. Insets show that p53 is not anymore upregulated at the otocyst stage in mutants. (**G,H**) The small-sized E10.5 otocysts of mutants show expanded Sox2-positive domain and limited neuroblast delamination. A rudimentary cochleovestibular ganglion is seen. (**I,J**) Pax2-positive domain is prominently present in both mutant and control otocysts. (**E,G,I** and **F,H,J**) are from adjacent sections. Abbreviations: CVG, cochleovestibular ganglion; D, dorsal; HE, hematoxylin; M, medial; ov, otic vesicle; PH3, phosphorylated histone-3. Scale bar, shown in **J:** (**A–D**), 35 μm; (**E–J**), 85 μm, insets in (**E,F**), 165 μm.

At embryonic day 8.5 (E8.5), otic placodes had formed in *Mdm2*^*FM*/*FM*^*;Pax2-Cre* embryos, but they were abnormally small and filled with apoptotic cells, detected by cleaved caspase-3 immunostaining (Fig. 1A–F). The concomitant strong p53 upregulation suggested that the cell death was a consequence of p53 accumulation (Fig. 1G,H). However, a portion of placodal progenitors -apparently those spared from recombination and consequent p53-mediated cell death- invaginated and formed a vesicle at E9.0. These mutant vesicles were clearly smaller than those of littermate controls (Fig. 2A,B). Consistent with our suggestion that the otic vesicles of mutant mice were formed by non-recombined cells, the extent of apoptosis in E9.5 vesicles was comparable to that seen in controls (Fig. 2C,D). Comparable mitotic activity in E10.5 mutant and control otocysts, detected by phospho-histone 3 staining, pointed to our conclusion as well, as high levels of p53 would be expected to antagonize cell cycle activity (Fig. 2E,F). Importantly, mutant otocysts did not anymore show p53 upregulation (Fig. 2E,F; insets). Sox2 staining revealed that although neuroblasts delaminated from the otocyst epithelium at E10.5, the neuroblast population and otocyst size were reduced compared to controls (Fig. 2G,H). Also, even though Sox2 expression was regionalized in mutant otocysts, this domain was abnormally broad (Fig. 2G,H). Maintained Pax2 expression in the otocyst epithelium (Fig. 2I,J) and the lack of apoptosis of Pax2-positive cells (data not shown) further supported our conclusion that a part of placodal progenitors had escaped recombination in *Mdm2*^*FM*/*FM*^*;Pax2-Cre* embryos.

**Figure 3:**
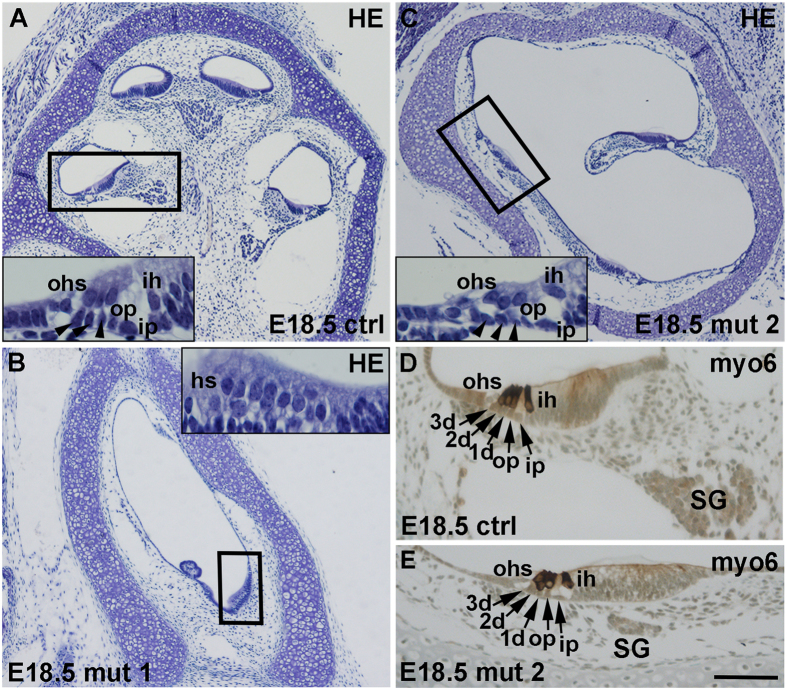
Strongly altered cochlear morphology of *Mdm2*^*FM*/*FM*^*;Pax2-Cre* mice at birth. (**A–C**) At E18.5, hematoxylin-stained sections show two examples of mutant cochleas with strong dysmorphogenesis. Boxed areas correspond to the higher magnification views shown in insets. Insets show varying degrees of cell organization in mutant organ of Corti. Arrowheads in insets denote Deiters’ cells. (**D,E**) Adjacent sections show organized pattern of hair cells, marked by myosin 6, and supporting cells (arrows). A rudimentary spiral ganglion is seen as well. Abbreviations: d, Deiters’ cell; HE, hematoxylin; hs, hair cells; ih, inner hair cell; ip, inner pillar cell; myo6, myosin 6; ohs, outer hair cells; op, outer pillar cell; SG, spiral ganglion. Scale bar, shown in **E:** (**A–C**), 200 μm; insets in (**A–C**), 25 μm; (**D,E**), 55 μm.

*Mdm2*^*FM*/*FM*^*;Pax2-Cre* mice did not survive beyond birth. At E17.5 and E18.5, these mice showed highly dysmorphic cochleas, a phenotype seen in all individuals analyzed (data not shown, Fig. 3A–C). Despite the dramatically altered global morphogenesis, cochleas contained restricted areas of sensory epithelium where a surprisingly normal organization of hair cells and SCs was seen (Fig. 3A–E; insets). In addition, a few specimens had developed tiny islands of the spiral ganglion (Fig. 3D,E). We confirmed that these limited sensory areas lacked detectable p53 immunoreactivity, similar to all other regions of the otic epithelium generated from non-recombined placodal progenitors (data not shown, Fig. 2E,F; insets). Thus, even though a large part of the earliest otic progenitors were lost in *Mdm2*^*FM*/*FM*^*;Pax2-Cre* mice, the remaining cells contributed to a nascent otocyst and, further, to a highly malformed cochlea.

### Neonatal auditory supporting cells are highly sensitive to the disruption of Mdm2/p53 interaction

We next focused on postnatal auditory SCs. Mdm2/p53 interaction was selectively abrogated in SCs by generating *Mdm2*^*FM*/*FM*^;*Fgfr3-iCre-ER*^*T2*^ mice. Prior studies have shown efficient *Fgfr3-iCre-ER*^*T2*^-mediated recombination in two types of auditory SCs, the pillar and Deiters’ cells, during the early postnatal life^17^. The first postnatal week is characterized by active differentiation of the cells of the organ of Corti. During the second postnatal week, signaling cascades regulating cell fates and differentiation are downregulated^18^. At these stages, SCs, located between the hair cell rows, mature and develop prominent actin and microtubule-based cytoskeletal structures. Concomitantly, fluid-filled spaces open within the organ of Corti, of which the tunnel of Corti is the most prominent^19^,^20^.

**Figure 4:**
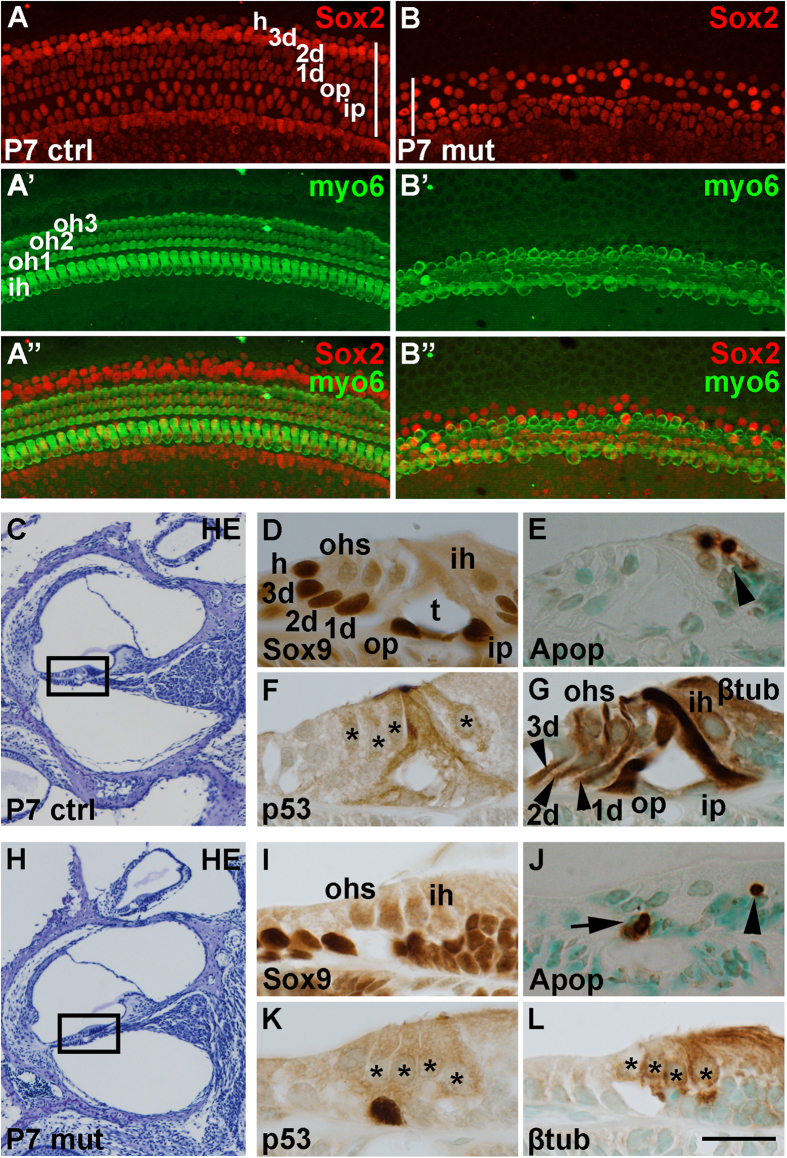
Disruption of Mdm2/p53 interaction triggers rapid death of neonatal auditory supporting cells, revealed in *Mdm2*^*FM*/*FM*^;*Fgfr3-iCre-ER*^*T2*^ mice. Tamoxifen-mediated recombination was induced at P0 and P1, and analysis performed at P7. Images are from the medial part of control and mutant cochleas. The rows of the different supporting cell (**A**) and hair cell types (**A’**) are marked. (**A,B”**) The mutant whole mount specimen shows the loss of Sox2-positive supporting cells, but maintained population of myosin 6-positive hair cells. The width of the supporting cell region is decreased (compare the bars spanning the width of this region in **A** and **B**). (**C**–**G**) A hematoxylin-stained cross-section through the control cochlea (**C**). The boxed area corresponds to the higher magnification views of the organ of Corti seen in adjacent sections (**D–G**). Sox9 marks supporting cells (**D**). ApopTag staining marks cell death that is seen in the greater epithelial ridge during the first postnatal week (arrowhead in **E**). Control specimen lacks nuclear p53 staining (**F**). β-tubulin antibody marks the prominent microtubule bundles in supporting cells (arrowheads in **G**). (**H–L**) Hematoxylin-staining shows unaltered global morphology of the mutant cochlea (**H**). The boxed area corresponds to the higher magnification views of the organ of Corti seen in adjacent sections (**I–L**). Sox9 staining reveals partial loss of supporting cells (**I**). ApopTag staining shows fragments of dying cells in the supporting cell area as well as in the greater epithelial ridge (arrow and arrowhead, respectively, in **J**). p53 is strongly expressed in the supporting cell nuclei (**K**). β-tubulin immunostaining shows the organ of Corti with perturbed cytoarchitecture (**L**). Asterisks mark hair cell nuclei. Abbreviations: Apop, ApopTag staining; β-tub, β-tubulin; d, Deiters’ cell; HE, hematoxylin; h, Hensen’s cell; ih, inner hair cell; ip, inner pillar cell; myo6, myosin 6; ohs, outer hair cells; op, outer pillar cell; t, tunnel of Corti. Scale bar shown in **L:** (**A,B”**), 50 μm; (**C,H**), 200 μm; (**D–G, I–L**), 15 μm.

After tamoxifen treatment at postnatal day 0 (P0) and P1, cochleas from *Mdm2*^*FM*/*FM*^;*Fgfr3-iCre-ER*^*T2*^ mice were analyzed at P4 and P7. A part of mutant mice had to be killed during this analysis period due to their worsened general health. At P4 (data not shown) and more prominently at P7 (Fig. 4A,B”), mutant animals displayed an organ of Corti with reduced width (lateral-to-medial) and height (basal lamina-to-lumenal surface), as assessed in whole mount surface specimens. SC loss was evident in these specimens. Quantitative analysis revealed that a significant part of SCs were rapidly lost following recombination and that this loss had progressed by P7 (P4: 38.0% ± 1.4 vs. P7: 47.4% ± 1.4). All hair cells, detected by myosin 6, were preserved in *Mdm2*^*FM*/*FM*^;*Fgfr3-iCre-ER*^*T2*^ mice (Fig. 4A’’). These data are consistent with prior data showing that *Fgfr3-iCre-ER*^*T2*^-mediated recombination does not target hair cells, with the exception of the most apical and basal parts of the cochlea that were not analyzed in the present study^17^.

As shown in paraffin sections at P7, global cochlear morphology was comparable between mutant and control mice (Fig. 4C,H). However, the morphology of the organ of Corti of mutant mice was abnormal due to the loss of Sox2-positive SCs (Fig. 4D,I). SC loss was seen throughout the cochlear duct (data not shown). The presence of ApopTag-positive profiles in the organ of Corti, detected in adjacent sections, pointed to apoptotic cell death (Fig. 4E,J). p53 accumulation in SC nuclei indicated that apoptosis was mediated by p53 (Fig. 4F,K). γH2AX was not upregulated in SCs of mutant cochleas, excluding the involvement of DNA damage in cellular death (data not shown). β-tubulin immunostaining revealed impaired morphological differentiation of SCs, likely associated with ongoing degeneration of these cells (Fig. 4G,L). These sections also revealed that hair cells were unaffected in mutant mice, in line with the results obtained with whole mount specimens (Fig. 4A’’). Together, our data demonstrates that high p53 sensitivity is maintained in the early postnatal, postmitotic progeny of the embryonic otic progenitors.

### Juvenile auditory supporting cells show increased resistance to the disruption of Mdm2/p53 interaction

**Figure 5:**
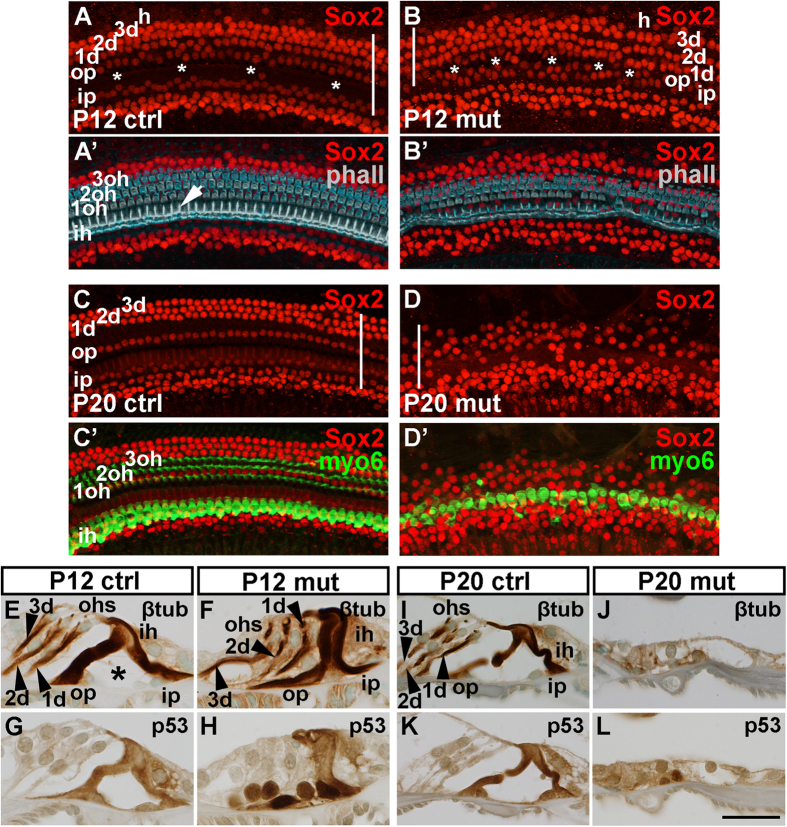
Disruption of Mdm2/p53 interaction causes delayed death of juvenile auditory supporting cells, revealed in *Mdm2*^*FM*/*FM*^;*Fgfr3-iCre-ER*^*T2*^ mice. Tamoxifen-mediated recombination was induced at P6 and P7, and analysis performed at P12 and P20. Images are from the medial part of control and mutant cochleas. Rows of the different supporting cell and hair cell types are marked. Asterisks mark the tunnel of Corti. (**A,B’**) At P12, whole mount specimen from a mutant shows that the Sox2-positive supporting cell population is largely present, but rows of these cells are to some extent disorganized (**B**). The width of the supporting cell region is decreased (compare the bars spanning the width of this region in **A** and **B**). Phalloidin strongly labels the actin cytoskeleton of the four hair cell rows and the apical processes of pillar cells (arrow in **A’**). This labeling reveals scattered loss of outer hair cells in the mutant specimen (**B’**). (**C–D’**) At P20, a large part of Sox2-positive supporting cells and myosin 6-positive outer hair cells are lost (**D,D’**). The width of the supporting cell region is decreased (compare the bars spanning the width of this region in **C** and **D**). Note that the inner hair cell row is preserved. (**E,F**) At P12, β-tubulin-stained sections show that Deiters’ (arrowheads) and especially pillar cells of the mutant cochlea lack a proper upright position and the tunnel of Corti (asterisk) between pillar cells is unopened. (**G,H**) At P12, p53 is upregulated exclusively in the supporting cell nuclei in the mutant organ of Corti. Control specimen lacks this nuclear staining. (**I,J**) At P20, β-tubulin staining reveals that the organ of Corti is replaced by a flattened epithelium. (**K,L**) At P20, a few p53-positive supporting cells are seen in the flattened epithelium of the mutant cochlea. Abbreviations: β-tub, β-tubulin; d, Deiters’ cell; h, Hensen’s cell; ih, inner hair cell; ip, inner pillar cell; myo6, myosin 6; oh, outer hair cell; op, outer pillar cell; phall, phalloidin. Scale bar shown in **L:** (**A–D’**), 50 μm; (**E–L**), 25 μm.

To investigate whether p53 sensitivity is maintained in juvenile SCs undergoing morphological maturation, tamoxifen was administered to *Mdm2*^*FM*/*FM*^;*Fgfr3-iCre-ER*^*T2*^mice at P6 and P7, and their cochleas were analyzed at P12 and P20. Only a small part of SCs were lost at P12 (Fig. 5A,B), but longer-term p53 accumulation resulted in the loss of the majority of SCs, as evidenced at P20 (Fig. 5C,D). When SC loss in juvenile (P6/P7) and neonatal (P0/P1) cochleas was quantitatively compared 6 days after recombination, juvenile SCs showed a highly significant increase in survival (juvenile: 6.3% ± 2.0 vs. neonatal: 47.4% ± 1.4). Thus, postmitotic SCs of *Mdm2*^*FM*/*FM*^;*Fgfr3-iCre-ER*^*T2*^mice die in an age-dependent manner.

Analysis of paraffin sections at P12 showed that the β-tubulin-positive apical processes of Deiters’ and pillar cells of mutant mice did not rise to an upright position, resulting in collapse of the tunnel of Corti (Fig. 5E,F). Adjacent sections revealed p53 upregulation in these cells (Fig. 5G,H). Most SCs were still present at P12, but a large part of them were lost by P20. At this stage, the organ of Corti had transformed into a flattened epithelium, both in the medial-to-lateral and basal-to-apical axes (Fig. 5I,J). p53 was upregulated in the remaining SCs (Fig. 5K,L). In addition to the specific nuclear staining, the p53 antibody produced non-specific cytoplasmic staining, especially in the juvenile and adult pillar cells that possess prominent cytoskeletal structures. This non-specificity was concluded from the cytoplasmic, but not nuclear p53 staining in *p53*-depleted SCs (*p53*^*fl*/*fl*^*;Pax2-Cre* mice; Supplementary Fig. S1). Despite the fact that outer hair cells of *Mdm2*^*FM*/*FM*^;*Fgfr3-iCre-ER*^*T2*^ mice did not recombine and upregulate p53 (Fig. 5G,H,K,L), most of these cells were lost by P20 (Fig. 5A’’). Taken together, p53 accumulation arrests maturation of juvenile SCs, ultimately leading to their death. It appears that outer hair cell death is a secondary event, caused by degeneration of adjacent, recombined SCs.

### Adult auditory supporting cells are resistant to the disruption of Mdm2/p53 interaction

To study the p53 response of mature auditory SCs, tamoxifen-mediated recombination was induced in *Mdm2*^*FM*/*FM*^;*Fgfr3-iCre-ER*^*T2*^mice at P49 and P50. The efficiency of recombination at adulthood was confirmed by generating *Ai14 (tdTomato*);*Fgfr3-iCre-ER*^*T2*^ mice. Based on red-fluorescent-protein (RFP) immunohistochemistry on paraffin sections, the whole population of adult pillar and Deiters’ cells was recombined (Supplementary Fig. S2), consistent with previous studies using the same reporter mice at a younger age^17^.

In contrast to the early postnatal SCs, SCs of adult *Mdm2*^*FM*/*FM*^;*Fgfr3-iCre-ER*^*T2*^mice survived and failed to show morphological defects, as assessed 6 days after the induction of recombination, at P55 (Supplementary Fig. S3). Despite this unaltered morphology, p53 was strongly upregulated in the adult SCs (Supplementary Fig. S3C,D; insets). These findings point to increased p53 resistance of adult compared to early postnatal SCs. Due to the worsened general health of these mutant animals, the long-term fate of adult SCs with p53 accumulation could not be followed.

### p53 upregulation is detrimental to hair cells and supporting cells of the late-embryonic cochlea

**Figure 6:**
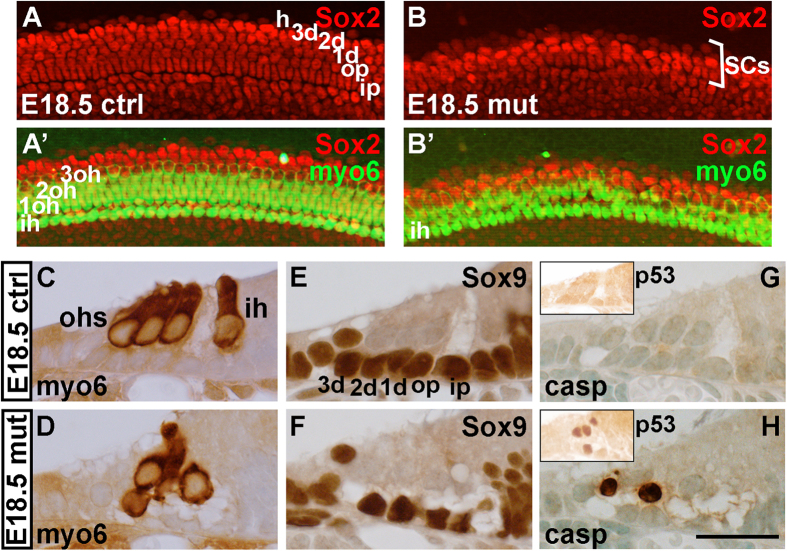
Disruption of Mdm2/p53 interaction is lethal to late-embryonic auditory hair cells and supporting cells, revealed *Mdm*^*FM*/*FM*^;*Fgfr3-iCre-ER*^*T2*^ mice. Tamoxifen-mediated recombination was induced at E13.5 and E14.5, and analysis performed at E18.5. (**A,B’**) Whole mount specimens show the loss of both myosin 6-positive hair cells and Sox2-positive supporting cells. Width of the organ of Corti is decreased in the mutant specimen (bracket in **B**). (**C–F**) Immunohistochemistry on paraffin sections shows degeneration of hair cells and supporting cells in the mutant organ of Corti. (**G,H**) The apoptotic marker cleaved caspase-3 is seen in the mutant, but not control organ of Corti. Insets show that p53 is upregulated only in the mutant organ of Corti. Abbreviations: casp, cleaved caspase-3; d, Deiters’ cell; ih, inner hair cell; ip, inner pillar cell; myo6, myosin 6; ohs, outer hair cells; op, outer pillar cell. Scale bar shown in **H:** (**A–B’**), 50 μm; (**C–H**), 20 μm; insets in (**G,H**), 55 μm.

In order to study p53 sensitivity of the cells of the late-embryonic organ of Corti, *Mdm2*^*FM*/*FM*^*;Fgfr3-iCre-ER*^*T2*^ mice received tamoxifen at E13.5 and E14.5, and their cochleas were analyzed at E18.5. This mid-embryonic recombination paradigm enabled us to study the consequences of *Mdm2* ablation in both hair cells and SCs, as described previously^21^. As cell cycle exit of precursor cells occurs at the time of recombination, these experiments focused on the response of postmitotic, early-differentiating cells to *Mdm2* ablation. Consistent with the known sensitivity of developing auditory hair cells to pharmacological p53 accumulation *in vitro*^22^, hair cells from *Mdm2*^*FM*/*FM*^*;Fgfr3-iCre-ER*^*T2*^ mice showed a strong death-prone phenotype (Fig. 6A’). Also SCs were lost in these cochleas (Fig. 6A,B,E,F), similar as in neonatal cochleas (Fig. 4B). Cell death was confirmed by cleaved caspase-3 staining (Fig. 6G,H). p53 upregulation in adjacent sections suggests that SC death is caused by p53 induction (Fig. 6G,H; insets). Altogether, neither SCs nor hair cells tolerate p53 upregulation during their differentiation.

### Pharmacological p53 upregulation abrogates supporting cell survival in an age-dependent manner

**Figure 7:**
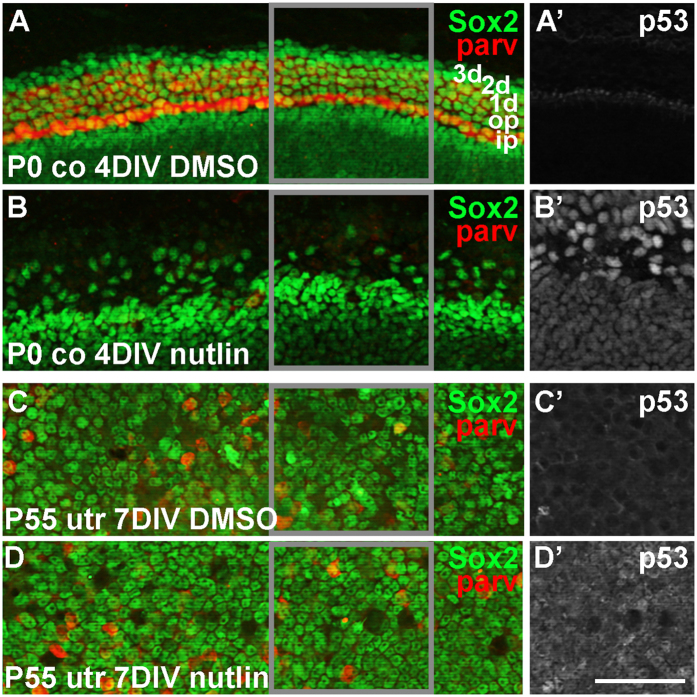
Nutlin3-triggered abrogation of Mdm2/p53 interaction kills inner ear supporting cells in an age-dependent manner *in vitro*. (**A,B**) Nutlin3, but not DMSO, triggers the loss of Sox2-positive supporting cells in P0 cochlear explants after 4 DIV. All parvalbumin-positive hair cells are also lost. Gray-boxed areas correspond to the views shown in **A’** and **B’**. (**A’,B’**) p53 is upregulated only upon nutlin3 treatment. (**C,D**) Nutlin3- and DMSO-treated P55 utricular explants show comparable amounts of Sox2-positive supporting cells and parvalbumin-positive hair cells after 7 DIV. Gray-boxed areas correspond to the views shown in **C’** and **D’**. (**C’,D’**) p53 is upregulated only in the nutlin3-treated utricular explant. Abbreviation: co, cochlea; parv, parvalbumin; utr, utricle. Scale bar shown in (D’), 50 μm.

The small molecule compound nutlin3 blocks Mdm2/p53 interaction, triggering p53 upregulation^23^. By preparing explants of the neonatal auditory sensory epithelium and maintaining them for 4 days *in vitro* (DIV) together with nutlin3, we first confirmed p53 upregulation in the explants (Fig. 7A’’). A large part of SCs were lost after 4 DIV and all of them had disappeared by 7 DIV (Fig. 7A,B, data not shown). There was a total absence of hair cells already at 4 DIV (Fig. 7A,B). The nutlin3 response of mature auditory cells could not be studied due to the difficulties in maintaining the adult organ of Corti in culture conditions. Therefore, we prepared explants of the adult utricle, one of the balance organs that tolerates culture conditions. The utricle contains hair cells and SCs that resemble those of the cochlea. P55 utricular explants from wildtype mice were maintained for 7 DIV with continuous nutlin3 exposure. Despite p53 induction, SCs in these adult cultures were viable (Fig. 7C,D’). Interestingly, also hair cells were present in amounts comparable to those seen in vehicle-treated, age-matched specimens (Fig. 7C,D). Altogether, these data are consistent with our *in vivo* data demonstrating age-related decrease in the sensitivity of SCs to the disruption of Mdm2/p53 interaction.

### *p53* inactivation is dispensable for cell cycle re-entry of auditory supporting cells

**Figure 8:**
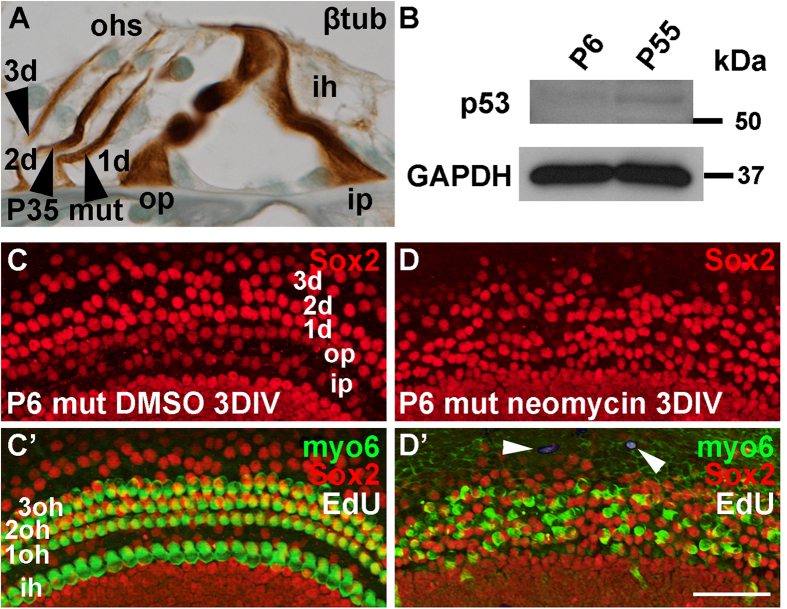
Supporting cells do not re-enter the cell cycle in cochlear explants from *p53*^*fl*/*fl*^*;Pax2-Cre* mice. (**A**) β-tubulin-stained section shows unaltered morphology of the organ of Corti of a mutant mouse at P35 (compare with Supplementary Fig. S3E). (**B**) Western blotting shows that p53 is weakly expressed in control cochleas both at P6 and P55. GAPDH was used as a loading control. (**C,C’**) DMSO-treated explant from a P6 mutant mouse displays a normal complement of Sox2-positive supporting cells and myosin 6-positive hair cells after 3 DIV. Both cell types are EdU-negative. (**D,D’**) Neomycin-challenged explant from a P6 mutant mouse lacks EdU-positive cells in the organ of Corti after 3 DIV. Note the presence of a few EdU-labeled cells outside this organ (arrowheads). Abbreviations: β-tub, β-tubulin; d, Deiters’ cell; myo6, myosin 6; ih, inner hair cell; ip, inner pillar cell; oh, outer hair cell; op, outer pillar cell. Scale bar shown in **D:** (**A**), 15 μm; (**C,D’**), 50 μm.

To study the effects of *p53* inactivation, we generated *p53*^*fl*/*fl*^*;Pax2-Cre* mice where *p53* is conditionally inactivated in the Pax2-positive otic progenitor cells from the placodal stage onwards^16^,^24^. These mice were viable and lacked phenotypic changes during development and adulthood, as assessed at birth and at P35 (data not shown; Fig. 8A). Despite the lack of an altered phenotype, Western blotting assays showed that p53 is expressed in low levels in the control cochlear tissue at P6 and P55 (Fig. 8B).

Next, we studied whether the loss of *p53* facilitates cell cycle re-entry of the normally quiescent SCs. Cochlear explants were prepared from *p53*^*fl*/*fl*^*;Pax2-Cre* mutant and control mice at P6, and maintained for 3 DIV. Neither mutant nor control explants supplemented with the replication marker EdU showed SC proliferation (Fig. 8C,C’, data not shown). To test whether *p53* inactivation facilitates β-catenin-mediated proliferative plasticity^25^,^26^, the glycogen synthase kinase-3α/β (GSK-3α/β) inhibitor BIO^27^ was added to P6 cultures to activate β-catenin. However, BIO treatment did not abrogate SC quiescence, neither in control nor mutant explants, based on the lack of EdU labeling (data not shown). We conclude that the absence of p53 does not confer proliferative plasticity to immature SCs.

Hair cell loss has been shown to stimulate cell cycle re-entry of neonatal SCs^28^. To test whether *p53* loss extends this proliferative response to the juvenile stage, P6 explants were prepared from *p53*^*fl*/*fl*^*;Pax2-Cre* mutant and control mice, and the explants were exposed to neomycin, an aminoglycoside antibiotic that kills hair cells. After neomycin challenge for 24 h, explants were maintained for additional 48 h in EdU-supplemented medium. Even though most hair cells were lost in these cultures, no EdU-positive SCs were detected, neither in control nor mutant explants (Fig. 8C,D’). Furthermore, although previous studies have reported that *p53* inactivation confers hair cell protection against ototoxic agents^29^,^30^, we could not see a difference in the extent of hair cell loss between neomycin-exposed mutant and control explants (data not shown).

### *p53* inactivation does not boost supporting-cell-to-hair cell transdifferentiation

**Figure 9:**
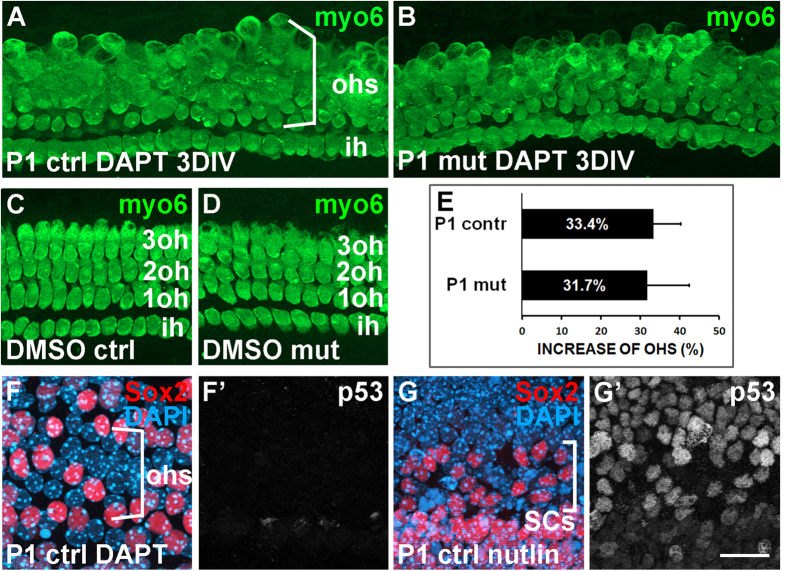
Supporting cell transdifferentiation into hair cells is not enhanced in cochlear explants from *p53*^*fl*/*fl*^*;Pax2-Cre* mice. (**A–D**) DAPT-treated explants from P1 mutant and control cochleas show accumulation of myosin 6-positive outer hair cells, as opposed to the organized rows of hair cells in DMSO-treated explants, assessed after 3 DIV. (**E**) Quantification reveals no significant difference in the amount of extra hair cells between the genotypes. (**F,G’**) p53 is not upregulated in the DAPT-treated control explant, as opposed to the nutlin3-treated control explant, assessed after 4 DIV. Sox2 marks supporting cell nuclei and DAPI cell nuclei. Note that a part of cells is lost in the nutlin3-treated specimen. Abbreviations: ih, inner hair cell; myo6, myosin 6; oh, outer hair cell. Scale bar shown in (G’), 20 μm.

Neonatal SCs can transdifferentiate into hair cells in response to Notch inhibition^31^. This transdifferentiation capacity is prominent neonatally, but abruptly declines at around P6^18^. As p53 is known to limit somatic cell reprogramming, we next studied whether *p53* inactivation increases transdifferentiation efficiency and whether it extends the period of reprogramming plasticity beyond the neonatal stage. Transdifferentiation was triggered in P1 and P6 cochlear explants from *p53*^*fl*/*fl*^*;Pax2-Cre* mutant and control mice. The γ-secretase inhibitor DAPT, an inhibitor of Notch signaling, was used to trigger transdifferentiation. DAPT-treated explants from both genotypes showed comparable amounts of supernumerary hair cells (Fig. 9A–E, data not shown). In line with these findings, p53 was not upregulated in DAPT-treated control explants (Fig. 9F,F’). In these experiments, nutlin3-treated explants were used as positive controls for p53 upregulation (Fig. 9G,G’). Thus, the absence of *p53* appears not to affect the molecular remodeling events that set the limits to the capacity of SCs to transdifferentiate into hair cells.

## Discussion

Cells have a complex machinery that maintains low levels of p53 under normal conditions. p53 is most commonly upregulated following abnormal proliferation and ensuing DNA damage, when it orchestrates the DNA damage response and DNA repair. If DNA damage remains unrepaired, p53 mediates cellular senescence or death^1^. Also certain developmental syndromes lead to p53 stabilization and activation^32^. In addition, p53 is induced in adult tissues upon a wide spectrum of stressful conditions and, thus, can also affect non-proliferating cells^1^. The postmitotic status of a cell is considered to increase resistance to elevated p53 levels. An interesting question is whether the cells that have exited the cell cycle, but are still undergoing differentiation or maturation respond to elevated p53 levels more like proliferating progenitor cells or like mature cells. These immature cells often show plasticity towards regenerative manipulations, such as stimulated cell cycle re-entry or cell fate conversion. It has been suggested that p53 upregulation limits the extent of these regenerative events and sets barriers to the regenerative capacity of adult cells^14^,^15^. Based on these considerations and the fact that auditory SCs serve as an attractive cell model in regeneration studies aiming to restore hearing loss, we found it important to study the response of these cells to increased and, on the other hand, suppressed p53 expression.

As an E3 ubiquitin ligase, Mdm2 mediates ubiquitination and proteasome-dependent degradation of p53^4^. We found that the inner ear cells respond to *Mdm2* abrogation by p53 upregulation and by phenotypic changes. Therefore, it can be concluded that these cells express p53 under normal conditions and that p53 levels are actively suppressed by Mdm2. We detected endogenous p53 in the cochlea by Western blotting assays, but not by immunohistochemistry. These results point to low levels of p53 in this tissue, consistent with the low abundance of p53 found in other organs^33^. These data reflect the key role of Mdm2 in preventing p53 accumulation under normal conditions.

In *Mdm2*^*FM*/*FM*^*;Pax2-Cre* mice, *Mdm2* was abrogated in the inner ear anlagen, the otic placode. Its progenitor cells upregulated p53, leading to rapid and extensive apoptosis. In the *Pax2-Cre* mouse line, *Cre* mRNA is induced in the early otic placode, but it is quickly downregulated^16^. Thereby, by using *Mdm2*^*FM*/*FM*^*;Pax2-Cre* mice, we were able to study what happens when the earliest otic progenitors are lost. It has been shown that a portion of placodal progenitors remain unrecombined in *Pax2-Cre* mice^16^. Most probably these unrecombined cells, that escaped *Mdm2* inactivation, gave rise to the small otic vesicles in *Mdm2*^*FM*/*FM*^*;Pax2-Cre* mice. The ultimate outcome of the extensive loss of early progenitors was a highly dysplastic inner ear, as assessed at birth. From the perspective of inner ear development it is interesting that an otic placode with strongly reduced numbers of progenitor cells can generate a vesicle. However, the vesicle was abnormally small, indicating that the early-occurring, extensive apoptosis cannot be later compensated by enhanced proliferation. These growth defects were linked with abnormalities in the expression of patterning molecules in the otocyst and, further, with impaired cochlear morphogenesis. Our results are in line with previous results on other embryonic tissues, showing that Mdm2/p53 abrogation and consequent p53 upregulation trigger severe pathologies^4^. Importantly, prior studies have shown that cell death, defects in organogenesis and embryonic lethality can be rescued by concomitant *p53* inactivation, indicating that p53 is a major target of Mdm2^5^,^6^.

The present results on *Mdm2*^*FM*/*FM*^*;Fgfr3-iCre-ER*^*T2*^ mice show that Mdm2 continues to be critical in regulating p53 levels in postmitotic, differentiating SCs. Following birth, Mdm2/p53 abrogation was induced exclusively in SCs in the cochlea. We found that the effects of p53 upregulation were distinctly age-dependent. During the first postnatal week, differentiating SCs were hypersensitive to p53, evidenced by their acute death upon p53 induction. During the second postnatal week, SCs undergoing structural maturation died only after a prolonged exposure to p53. Also adult SCs tolerated p53 accumulation surprisingly well. These age-dependent differences in p53 sensitivity were not due to differential recombination patterns, as both the early postnatal and adult SCs showed nearly 100% recombination efficiency, based on the analysis of *Ai14*(*tdTomato*)*;Fgfr3-iCre-ER*^*T2*^ reporter mice^17^ (present study). Consistent with our findings, late-embryonic neurons of the central nervous system die rapidly and in large amounts upon conditional *Mdm2* ablation^7^. In another study, the effects of inducible, global *Mdm2* inactivation were studied in various adult tissues. In line with our results, that study showed that tissues comprising postmitotic cells (“radio-insensitive tissues”) are more sensitive to p53 upregulation during the young adult compared to older, more mature stage^11^.

What confers increased p53 resistance to juvenile (second postnatal week in our study) and adult SCs compared to neonatal (first postnatal week) SCs? In *Mdm2*^*FM*/*FM*^*;Fgfr3-iCre-ER*^*T2*^ mice, cell death was not associated with activation of the DNA damage response, based on the absence of γH2AX induction. This result is not unexpected considering the postmitotic status of SCs. In adult cells in general, a large portion of chromatin is condensed and silenced. Accessibility of the p53 transcription factor to pro-apoptotic target genes, particularly to *Puma, Bax*, and *Noxa*, becomes limited along aging, resulting in weakening of its apoptosis-promoting function^34^. In the mouse kidney, binding of p53 to *Puma* is significantly decreased in mature compared to young cells^11^. In auditory SCs of the mouse, signaling cascades regulating differentiation are downregulated by the end of the first postnatal week, shown, for example, in the case of Notch signaling^18^. Epigenetic mechanisms might be involved in limiting transcriptional activation of differentiation promoting genes by Notch signaling. It has also been suggested that the proneural gene *Atoh1* is epigenetically silenced in auditory SCs at the end of the first postnatal week^35^. In line with these data, epigenetic mechanisms that promote heterochromatinization at the loci of pro-apoptotic genes might slow down their transcriptional activation by p53 and thereby cause delayed rather than acute cell death. Juvenile SCs of *Mdm2*^*FM*/*FM*^*;Fgfr3-iCre-ER*^*T2*^mice displayed halted differentiation before their death, indicating that it took longer time for the upregulated p53 to execute cell death. Also, p53 was acutely and strongly upregulated in SCs in response to *Mdm2* abrogation, despite delayed death of these cells. This implies that the age-dependent decline in p53 responsiveness is due to mechanisms downstream of p53 expression.

Inner ears of *p53*-inactivated mice (*p53*^*fl*/*fl*^*;Pax2-Cre*) failed to show gross developmental abnormalities, consistent with the unaltered phenotype of several *p53*-inactivated tissues thus far studied^36^. However, p53 suppression has been shown to increase regeneration efficiency. For example, p53 silencing enhanced fibroblast conversion into neurons and it was suggested that p53 is a major gatekeeper in the maintenance of the existing transcription network of the cell^37^,^38^. The reprogramming intervention in the cochlea, transdifferentiation of SCs into outer hair cells, can be induced by Notch inhibition, mainly during the first postnatal week^18^,^31^. However, we did not see enhanced transdifferentation in DAPT-treated cochlear explants from *p53*^*fl*/*fl*^*;Pax2-Cre* mice. Correspondingly, DAPT-treated wildtype explants did not show p53 accumulation. This result was not actually surprising, since SC-to-hair cell transdifferentiation triggered by Notch inhibition is not associated with cell death or cell cycle re-entry^39^. If this would be the case, DNA damage signaling would be activated, leading to p53 induction.

Could endogenous p53 restrict proliferative plasticity of quiescent auditory SCs? Critical regulators of cell cycle exit and the maintenance of the postmitotic state are the cyclin-dependent kinase inhibitors (CKIs) of the core cell cycle machinery. CKI inactivation has been shown to trigger ectopic replication of SCs during the early postnatal life^40^. As p53 is not directly involved in the maintenance of the postmitotic state^1^, it was not surprising that *p53*-depleted SCs failed to show unscheduled proliferation *in vivo* and *in vitro. p53* inactivation has, nevertheless, been shown to potentiate cell cycle re-entry of quiescent cells when applied in combination with exogenous mitogens, shown in the case of Muller glia in retinal cultures^41^. We did not see this effect in the early postnatal cochlear explants supplemented with the GSK-3α/β inhibitor BIO, neither in explants from *p53*^*fl*/*fl*^*;Pax2-Cre* nor control mice. BIO activates mitogenic Wnt/β-catenin signaling, a pathway whose activation has been shown to stimulate cell cycle re-entry of neonatal SCs^25^,^26^,^27^. As also aminoglycoside-induced hair cell death failed to activate SC proliferation in explants from *p53*^*fl*/*fl*^*;Pax2-Cre* mice, we conclude that *p53* loss does not facilitate the capacity of SCs to cell cycle re-entry or transdifferentiation. These findings do not contradict with our previous results showing that p53 is upregulated in the early postnatal SCs forced to re-enter the cell cycle^42^. It is known that unscheduled proliferation and consequent DNA damage trigger p53 upregulation, leading to cell cycle arrest that is often accompanied by apoptosis^43^. In our previous work, we showed that in response to oncogenic cyclin D1 overexpression, juvenile auditory SCs re-enter the cell cycle, but they have difficulties in progression into mitosis and in clonal expansion^42^.

Our results on nutlin3-treated explants support the data obtained with *Mdm2*^*FM*/*FM*^*;Fgfr3-iCre-ER*^*T2*^ mice, showing the death-prone phenotype of developing SCs following p53 upregulation. The *Fgfr3-iCre-ER*^*T2*^ mouse line enabled us to abrogate *Mdm2* during late-embryogenesis in auditory hair cells as well. We found that disruption of Mdm2/p53 interaction *in vivo* is detrimental to differentiating hair cells, consistent with prior studies showing the sensitivity of developing auditory hair cells to nutlin3 in explant cultures^22^. Certain human developmental syndromes cause unrestrained p53 activity, yet with a less severe phenotype and with delayed lethality compared to *Mdm2* ablation, apparently due to less dramatic increase in p53 levels^32^. Interestingly, some of these syndromes have been shown to include inner ear malformations or hearing loss, particularly the CHARGE syndrome^32^. This syndrome has been modeled in mice and the animals show defects in the morphogenesis of the vestibular labyrinth^32^,^44^,^45^,^46^. Based on our results, impaired survival of hair cells and SCs of the cochlea might be included in the spectrum of CHARGE phenotypes and perhaps also in some other developmental syndromes with unrestrained p53 activity. In conclusion, our results demonstrate an important role for the Mdm2/p53 interaction in ensuring survival of both proliferating progenitor cells and differentiating cells of the auditory organ.

## Methods

### Mice

*Mdm2*^*FM*/*FM*^*;Pax2-Cre* mice were generated by crossing floxed *Mdm2* mice (*Mdm2*^*FM*/*FM*^)^12^ with *Pax2-Cre* mice^16^. *Mdm2*^*FM*/*FM*^ mice were used as littermate controls. At least 4 mutant and 4 control embryos were analyzed per age (E8.5, E9.0, E9.5, E10.5, E17.5, E18.5). Both ears of each animal were analyzed.

*Mdm2*^*FM*/*FM*^*;Fgfr3-iCre-ER*^*T2*^ mice were obtained by crossing *Mdm2*^*FM*/*FM*^mice with *Fgfr3-iCre-ER*^*T2*^mice^47^. *Mdm2*^*FM*/*FM*^mice were used as littermate controls. Analysis was performed at E18.5 (tamoxifen at E13.5 and E14.5, *n* = 2 mutant and *n* = 2 control mice), at neonatal stages (P4, P7) (tamoxifen at P0 and P1, *n* = 4 mutant and *n* = 4 control mice), at juvenile stages (P12, P20) (tamoxifen at P6 and P7, *n* = 5 mutant and *n* = 8 control mice) and at adulthood (P55) (tamoxifen at P49 and P50, *n* = 3 mutant and *n* = 5 control mice). Both ears of each animal were analyzed.

*p53*^*fl*/*fl*^*;Pax2-Cre* mice were obtained by crossing floxed *p53* mice (*p53*^*fl*/*fl*^)^24^ (Jackson Laboratory, B6.129P2-*Trp53*^*tm1Brn*^/J) with *Pax2-Cre* mice^16^. *p53*^*fl*/*fl*^ mice were used as littermate controls. Both ears of 3 mutant and 3 control mice were analyzed per age (P0 and P35). The specificity of the p53 antibody was validated in cochleas from *p53*^*fl*/*fl*^*;Pax2-Cre* mice at P2 and P50.

To study the pattern of *iCre*-mediated recombination in the mature cochlea, *Fgfr3-iCre-ER*^*T2*^ mice were bred with the *ROSA26tm14*(*CAG-tdTomato) Cre*-conditional reporter mice (Jackson Laboratory). *Ai14*(*tdTomato*) transgene expression was detected by RFP immunohistochemistry. Both ears of 2 *Ai14*(*tdTomato*)*;Fgfr3-iCre-ER*^*T2*^mice receiving tamoxifen at P49 and P50 were analyzed at P55.

All mouse lines were maintained in a mixed background and both females and males were used in the analysis. Timed pregnancies were established by the detection of vaginal plug, taking the morning of plug observation as E0.5. The day of birth was considered as P0. Genotyping was performed as previously described^12^,^16^,^24^,^47^. All animal work has been conducted according to relevant national and international guidelines. Approval for animal experiments has been obtained from the local Laboratory Animal Centre of University of Helsinki (permission number KEK14-001) and the National Animal Experiment Board (permission number ESAVI/4606/04.10.07/2016).

### Induction of *iCre*-mediated recombination

*Mdm2*^*FM*/*FM*^*;Fgfr3-iCre-ER*^*T2*^ and control littermate mice were intraperitoneally injected with tamoxifen^17^ (Sigma-Aldrich) at 2 consecutive days: at E13.5 and E14.5 (3 mg per pregnant mother), at P0 and P1 (50 *μ*g/g body weight), at P6 and P7 (50 *μ*g/g body weight) and at P49 and P50 (200 *μ*g/g body weight). This latter paradigm was also used for *Ai14*(*tdTomato*)*;Fgfr3-iCre-ER*^*T2*^mice.

### Histological sections

Between E8.5 and E10.5, *Mdm2*^*FM*/*FM*^*;Pax2-Cre* mutant and control mice were fixed with 4% paraformaldehyde (PFA) for 6 h. At E17.5 and E18.5, dissected inner ears were immersed in PFA overnight. Postnatal cochleas were perilymphatically fixed and immersed in PFA overnight, and decalcified in 0.5 M EDTA, pH 7.5. Specimens were embedded in paraffin and cut to 5-μm-thick sections. Epitopes were unmasked by microwave boiling (900 W) in 10 mM citrate buffer, pH 6.0, for 10 min. Sections were incubated with primary antibodies in PBS containing 0.25% Triton-X-100 (PBS-T) and 10% normal serum for 48 h at +4 °C. The following primary antibodies were used: rabbit polyclonal Pax2 (1:1000, Zymed/Thermo Fisher Scientific), rabbit polyclonal p53 (1:400, Novocastra; NCL-p53-CM5p), rabbit monoclonal cleaved caspase-3 (1:250, Cell Signaling Technology), rabbit polyclonal β-tubulin (1:1000, Abcam), rabbit polyclonal RFP (1:500, Rockland Immunochemicals), rabbit polyclonal Sox9 (1:3000, Millipore), mouse monoclonal γH2AX (1:500, Millipore), rabbit polyclonal myosin 6 (1:3000, Proteus Biosciences), goat polyclonal Sox2 (1:3000, Santa Cruz Biotechnology) and mouse monoclonal PH3 (1:250, Cell Signaling Technology). Detection was done using the Vectastain Elite ABC and Mouse-on-Mouse Elite Peroxidase kits and the DAB Peroxidase Substrate kit (all from Vector Laboratories). Apoptag Peroxidase *In Situ* Apoptosis Detection Kit (Millipore) was used to detect DNA fragmentation. Sections were counterstained with methyl green and mounted in Permount (Fisher Scientific). A part of the consecutive sections was stained with hematoxylin (Shandon Instant Hematoxylin, Thermo Fisher Scientific).

### Whole mount specimens

Explants were fixed in 4% PFA for 10 min and permeabilized in PBS-T. Double- and triple-immunofluorescence was performed in PBS-T and 10% normal donkey serum for 48 h at +4 °C, using the following antibodies: rabbit polyclonal myosin 6 (1:500), goat polyclonal Sox2 (1:1000), rabbit polyclonal p53 (1:500) and mouse parvalbumin (1:2000, Swant). Secondary antibodies conjugated to Alexa Fluor 488, 568, 594 and 647 were used for visualization. Following antibody incubations, filamentous actin filaments were visualized using Alexa Fluor 647-labeled phalloidin (1:400). ProLong Gold anti-fade reagent was used for mounting (all from Molecular Probes/Invitrogen).

### Explant cultures

Explant cultures were established and maintained as previously described^42^. Cochleas at P0 and utricles at P55 were prepared from wild-type ICR mice, and maintained for 4 or 7 DIV with Nutlin3 (Cayman Chemicals) at the concentration of 10 μM. Explants treated with 0.1% DMSO were used as controls (*n* = 10 for each age and compound).

Cochlear explants were prepared from *p53*^*fl*/*fl*^*;Pax2-Cre* mutant and littermate control mice at P6 (*n* = 6 explants of each genotype), and maintained for 3 DIV with BIO ((2′Z,3′E)-6-bromoindirubin-3′-oxime; Calbiochem) at the concentration of 10 μM. Explants treated with 0.1% DMSO were used as controls (*n* = 6 for each genotype and compound). Cultures were pulsed every 24 h with 5 μM EdU (5-ethynyl-2′-deoxyuridine; Molecular Probes/Invitrogen).

Cochlear explant cultures were established from *p53*^*fl*/*fl*^*;Pax2-Cre* mutant and littermate control mice at P6, and treated with neomycin (Sigma-Aldrich) at the concentration of 500 μM for 24 h. Neomycin was substituted by PBS in control explants (*n* = 6 for each genotype and compound). Cultures were maintained for 2 additional days, pulsing every 24 h with 5 μM EdU.

Explant cultures were established from *p53*^*fl*/*fl*^*;Pax2-Cre* mutant and littermate control mice at P1 and P6, and maintained for 3 DIV in a medium supplemented with DAPT (N-[N-(3,5-difluorophenacetyl)-L-alanyl]-S-phenylglycine t-butyl ester, Sigma-Aldrich) at the concentration of 10 μM. This medium was changed every 24 h. Explants treated with 0.1% DMSO were used as controls (*n* = 6 for each genotype, age and compound). Specimens were stained for EdU, using the Click-iT EdU Alexa Fluor 555 Imaging Kit according to manufacturer’s instructions (Molecular Probes/Invitrogen). ProLong Gold was used for mounting.

### Immunoblotting

Six cochleas were dissected from control mice (*p53*^*fl*/*fl*^) at P6 and P55, and snap-frozen in liquid nitrogen. Cochleas comprised the otic capsule and the cochlear duct. Tissues were homogenized in lysis buffer (300 mM NaCl, 100 mM Tris-HCl, pH 7.5, 4 mM EDTA, 2% BSA, 0.2% Triton, 2 mM PMSF, 1 mM sodium orthovanadate, Protease Inhibitor Cocktail (Sigma)), incubated on ice for 1 h and centrifuged at 12000 rpm for 15 min at +4 °C. Protein samples (30 μg of each) were resolved by SDS-PAGE and proteins were transferred to a nitrocellulose membrane. Immunoblot analysis was performed using rabbit polyclonal p53 (1:1000, Novocastra; NCL-p53-CM5p) and mouse GAPDH (glyceraldehyde 3-phosphate dehydrogenase, 1:3000, Millipore) antibodies.

### Imaging

Paraffin sections were imaged with the BX61 microscope equipped with UPlanApo 4x, 10x, 20x and 60x objectives. Images were acquired through the DP73 CCD color camera and CellSens software (all from Olympus). Whole mount specimens were imaged under epifluorescence illumination using the Axio Imager.M2 microscope equipped with PlanApo 20x and 40x objectives and with Apotome 2 structured illumination slider (all from Zeiss). Images were acquired with the black and white CMOS camera (Hamamatsu ORCA Flash 4.0 V2) and ZEN 2 software (Zeiss). Images were processed using Adobe Photoshop CS6 (Adobe Systems).

### Statistical analysis

To determine the extent of cellular loss in the cochleas of *Mdm2*^*FM*/*FM*^*;Fgfr3-iCre-ER*^*T2*^ mice, Sox2-positive SCs and myosin 6-positive outer hair cells were counted from three 20-inner-hair-cell-width areas from the medial cochlear coil. Data are shown as the average with s.e.m. of the percentage of lost SCs and outer hair cells. To determine the amount of supernumerary outer hair cells in DAPT-treated cochlear explants from *p53*^*fl*/*fl*^*;Pax2-Cre* and control mice, myosin 6-positive cells were counted from a 20-inner-hair-cell-width area in the medial coil. Data are shown as the average with s.e.m. of the percentage of extra outer hair cells. Three to five explants were used for each quantification. Two-tailed Student’s t test was used for statistical analysis. Values were regarded as highly significant at *p* < 0.005.

## Additional Information

**How to cite this article:** Laos, M. *et al*. Indispensable role of Mdm2/p53 interaction during the embryonic and postnatal inner ear development. *Sci. Rep.* **7**, 42216; doi: 10.1038/srep42216 (2017).

**Publisher's note:** Springer Nature remains neutral with regard to jurisdictional claims in published maps and institutional affiliations.

## Supplementary Material

Supplementary Dataset 1
