# Supplementary material for: Indispensable role of Mdm2/p53 interaction during the embryonic and postnatal inner ear development
Source: Sci Rep. 2017 Feb 9;7:42216. doi: 10.1038/srep42216 (PMC5299844; doi:10.1038/srep42216)
Supplement: Supplementary Dataset 1 [file srep42216-s1.pdf]

Indispensable role of Mdm2/p53 interaction during the embryonic and postnatal inner ear development

Laos M, Sulg M, Herranen A, Anttonen T and Pirvola U*

Division of Physiology and Neuroscience, Department of Biosciences, University of Helsinki, 00014 Helsinki, Finland.

Corresponding author: Ulla Pirvola, e-mail: ulla.pirvola@helsinki.fi

Supplementary information

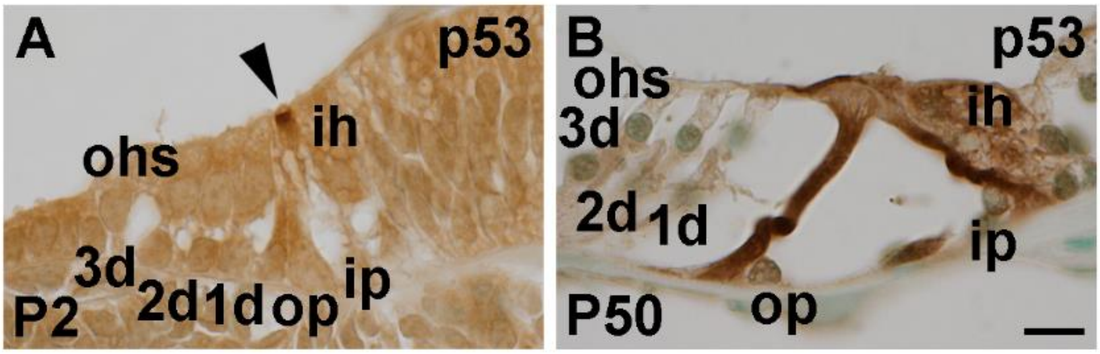

Supplementary Figure S1: Non-specific p53 staining in cochleas of *p53$^{fl/fl}$;Pax2-Cre* mice. Cochlear sections from *p53$^{fl/fl}$;Pax2-Cre* mice at P2 and P50 were stained with the p53 antibody. **(A)** At P2, early-differentiating head processes of pillar cells (arrowhead) of a mutant mouse show non-specific staining. **(B)** At P50, non-specific cytoplasmic staining is seen in the mature pillar cells and in inner hair cells of a mutant mouse. Abbreviations: d, Deiters' cell; ih, inner hair cell; ip, inner pillar cell; ohs, outer hair cells; op, outer pillar cell. Scale bar shown in B: 10 µm.

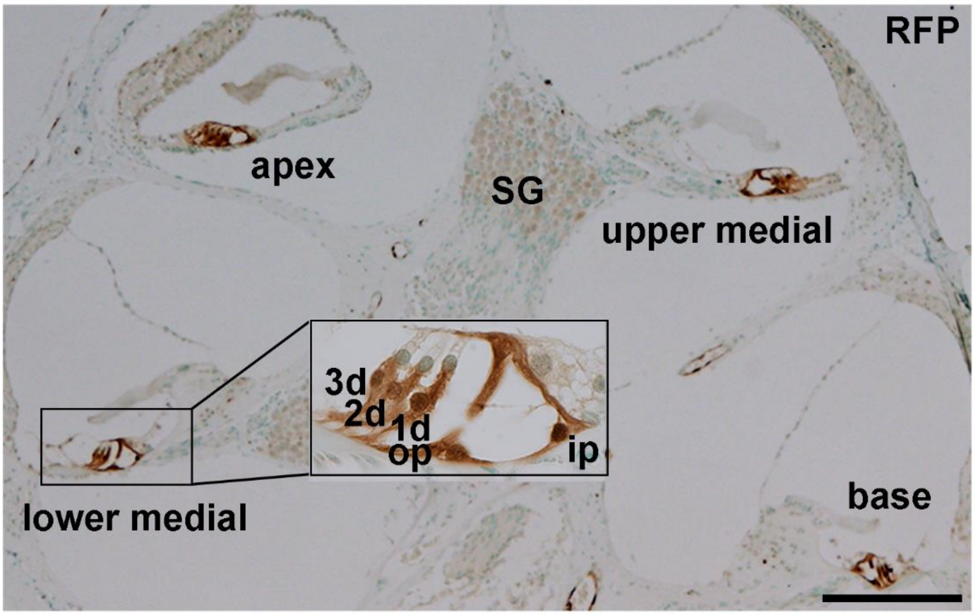

Supplementary Figure S2: Recombination pattern in the adult cochlea of *Ai14 (tdTomato);Fgfr3-iCre-ER^{T2}* mice. Tamoxifen-mediated recombination was induced at P49 and P50, and analysis performed at P55. Red-fluorescent-protein immunohistochemistry in paraffin sections shows selective recombination in Deiters' and pillar cells in the basal, medial and apical turns of the cochlear duct. Inset is from the medial coil. Abbreviations: d, Deiters' cell; ip, inner pillar cell; op, outer pillar cell; RFP, red fluorescent protein; SG, spiral ganglion. Scale bar, 200 µm; inset, 45 µm.

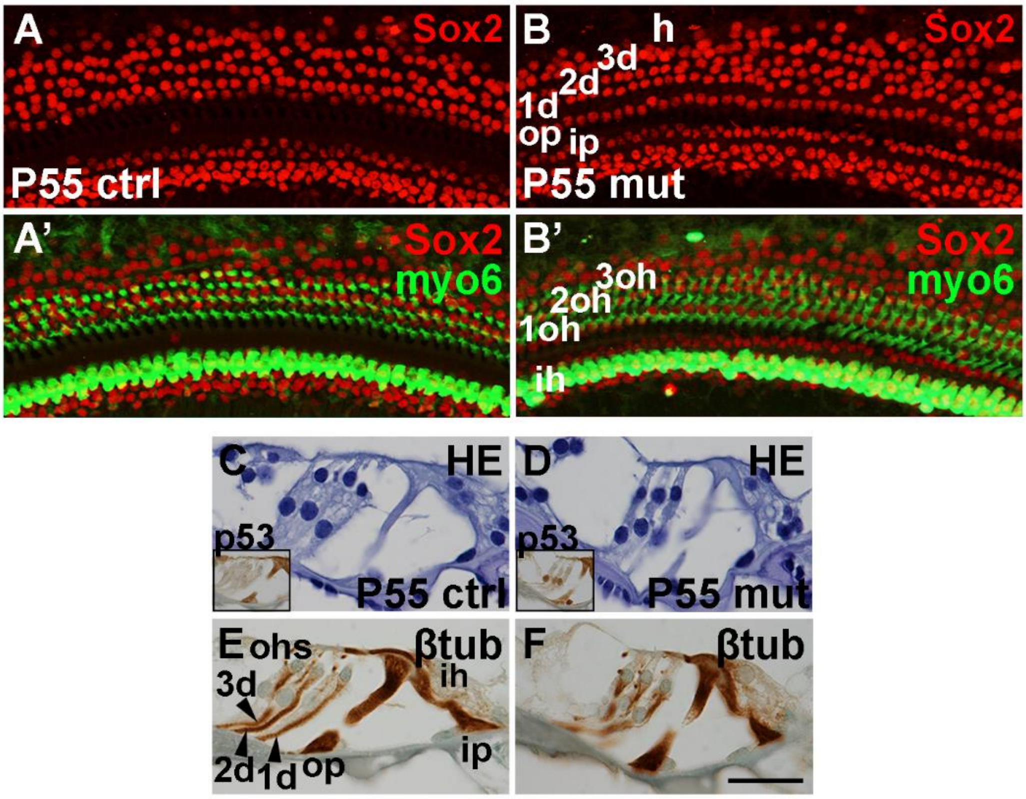

Supplementary Figure S3: Disruption of Mdm2/p53 interaction is tolerated by adult auditory supporting cells, revealed in *Mdm2$^{FM/FM}$;Fgfr3-iCre-ER^{T2}* mice. Tamoxifen-mediated recombination was induced at P49 and P50, and analysis performed at P55. **(A-B')** Whole mount specimens show a normal complement of Sox2-positive supporting cells and myosin 6-positive hair cells in the mutant organ of Corti. **(C-F)** Hematoxylin- and β-tubulin-stained sections lack morphological defects in the mutant organ of Corti. Insets in C,D show p53 accumulation in the supporting cell nuclei of a mutant, but not control specimen. Arrowheads in E mark the microtubule bundles in Deiters' cells. Abbreviations: β-tub, β-tubulin; d, Deiters' cell; HE, hematoxylin; h, Hensen´s cell; ih, inner hair cell; ip, inner pillar cell; myo6, myosin 6; oh, outer hair cell; op, outer pillar cell. Scale bar shown in F: A-B', 40 µm; C-F, 25 µm; insets in C,D, 70 µm.
